# Curcumin Regulates ERCC1 Expression and Enhances Oxaliplatin Sensitivity in Resistant Colorectal Cancer Cells through Its Effects on miR-409-3p

**DOI:** 10.1155/2020/8394574

**Published:** 2020-09-17

**Authors:** Wei Han, Hongli Yin, Hao Ma, Yi Wang, Desong Kong, Zhimin Fan

**Affiliations:** ^1^Chinese Medicine Modernization and Big Data Research Center, Nanjing Hospital of Chinese Medicine Affiliated to Nanjing University of Chinese Medicine, Nanjing University of Chinese Medicine, Nanjing 210022, China; ^2^General Surgery Department, Nanjing Hospital of Chinese Medicine Affiliated to Nanjing University of Chinese Medicine, Nanjing University of Chinese Medicine, Nanjing 210022, China; ^3^Proctology Department, Nanjing Hospital of Chinese Medicine Affiliated to Nanjing University of Chinese Medicine, Nanjing University of Chinese Medicine, Nanjing 210022, China

## Abstract

**Background:**

Oxaliplatin (L-OHP) resistance is a major obstacle to the effective treatment of colorectal cancer. The resistance mechanism(s) of colorectal tumors to L-OHP may be related to the regulation of *ERCC1* by cancer-expressed miRNAs, but no in-depth studies on the miRNAs that affect drug resistance have been performed. Curcumin (Cur) can reverse the drug resistance of cancer cells, but its effects on ERCC1 expression and miRNA profiles in colorectal cancer have not been studied.

**Methods:**

To study the regulation effect of curcumin on ERCC1 expression and its effects on miRNAs, the L-OHP-resistant colorectal cancer cell line HCT116/L-OHP was established. MTT assays were used to evaluate cell proliferation. Flow cytometry was used to investigate apoptotic induction. Western blot and RT-PCR analysis were used to evaluate the expression of drug-associated ERCC1, Bcl-2, GST-*π*, MRP, P-gp, and survivin.

**Results:**

HCT116//L-OHP cell lines were successfully established. The combination of L-OHP and curcumin could reduce L-OHP resistance *in vitro*. In addition, combination therapy inhibited the expression of ERCC1, Bcl-2, GST-*π*, MRP, P-gp, and survivin at the mRNA and protein level. Curcumin was found to inhibit ERCC1 through its ability to modulate miR-409-3p.

**Conclusion:**

Curcumin can overcome L-OHP resistance in colorectal cancer cells through its effects on miR-409-3p mediated ERCC1 expression.

## 1. Introduction

Colorectal cancer is a common tumor of the digestive tract that presents a threat to human life. Globally, the incidence of colorectal cancer ranks the third amongst all human cancers with mortality rates that rank the fourth amongst malignant tumors. In recent years, the incidence and mortality of colorectal cancer in China has shown a significant upwards trend [1]. Although surgical resection is the front-line treatment, chemotherapy is employed in advanced patients. L-OHP is a commonly used platinum-based chemotherapeutic drug that plays an important role in colorectal treatment [2]. Its main mechanism is to form interchain cross-links by covalently bonding with DNA double strands, inhibiting DNA replication and transcription to exert antitumor effects [3]. However, the long-term application of L-OHP leads to toxic side effects and drug resistance, the major causes of chemotherapy failure [4]. Excision repair cross-complementing gene (ERCC1) is key to the nucleotide excision repair (NER) system [5]. Recent studies suggest that the resistance to platinum drugs in cancer is related to high ERCC1 expression [6, 7]. A large number of studies have confirmed that miRNAs specifically bind to the 3′UTR of target mRNAs and regulate mRNA synthesis and protein expression [8]. miRNAs play an important role in the regulation of cell proliferation, differentiation, and apoptosis and participate in tumor development and progression [9]. In our previous studies, we found that variations in the ER321 3′-UTR region (rs3212986) are closely associated with L-OHP chemotherapy sensitivity, most likely due to its ability to regulate the transcription and translation of ERCC1 through miRNA binding [9]. Overcoming tumor cell multidrug resistance is an effective method to improve the efficacy of anticancer chemotherapy drugs. The identification of drugs that reverse tumor resistance has emerged as a research hot spot in the field of cancer therapy.

The low toxicity and high efficiency of traditional Chinese medicine has attracted intense research attention for the discovery of compounds that can reverse tumor resistance. Curcumin is a polyphenolic compound isolated from the rhizome of *Curcuma longa*, a traditional Chinese medicine [10]. Previous studies have confirmed that curcumin displays antitumor effects on a variety of cancers, including colorectal tumors [11–13]. In addition, curcumin has been shown to prevent tumor resistance [14]. Mehdi et al. [15] found that curcumin combined with 5-fluorouracil (5FU) can improve the chemotherapeutic effects of 5FU on colorectal cancer resistant strains by reducing mismatch repair ability. The current studies indicate that the resistance mechanisms of colorectal tumors to L-OHP may be related to the miRNA mediated regulation of ERCC1 [16], but no in-depth studies on the miRNAs that affect drug resistance and ERCC1 gene expression have been performed. Whether curcumin reverses the drug resistance of tumor cells through its ability to regulate miRNAs has not been defined.

In this study, we constructed a human colon cancer resistant L-OHP cell line HCT116/L-OHP and detected *β*-lymphoma 2 (Bcl2) and glutathione thiotransferase *π* (GST-*π*) in parental and drug-resistant cells. The expression of multidrug resistance-related protein (MRP), P-glycoprotein (P-gp), and the apoptosis-inhibiting gene survivin were assessed as markers of drug resistance. We assessed miRNA expression in drug-resistant cells and explored whether curcumin can reverse the resistance of human colon cancer cells through its ability to regulate specific miRNAs.

## 2. Materials and Methods

### 2.1. Establishment of HCT-116/L-OHP Human L-OHP-Resistant Cell Line

The human colon cancer cell line HCT-116 was purchased from the Shanghai Institute of Cell Bank Center (Shanghai, China). HCT-116 cells in the logarithmic growth phase were seeded in culture dishes at 5 × 10^5^ cells/mL. Cells were treated with 1 mg/L L-OHP (Jiangsu Hengrui Pharmaceutical Co., Ltd.) for 5–7 days. After 3 passages, the cells showed stable growth in the drug-containing medium. During this time, the L-OHP concentration was increased to 2 mg/L and gradually increased until the cells showed stable growth in 10 mg/L. The established cell lines were named HCT-116/L-OHP cells.

### 2.2. CCK-8 Analysis to Detect the HCT-116/L-OHP Resistance Index

HCT-116 and HCT-116/L-OHP cells were seeded into 96-well plates (3 × 10^3^ cells per well) for 24 h at 37°C in a CO_2_ incubator. Cells were treated with a range of concentrations of L-OHP (0, 1, 2, 5, 10, 20, and 50 mg/L) to a final volume of 200 *μ*L. After 48 h incubation, the culture medium was discarded and cells were treated with 200 *μ*L of culture medium containing 10 *μ*L of CCK-8 reagent (Japan Tongren Institute of Chemistry) for 2 h. The absorbance of each well was measured on a microplate reader at 450 nm.

### 2.3. CCK-8 Analysis to Detect the Inhibitory Effect of Curcumin on HCT-116 and HCT-116/L-OHP Cells

HCT-116 and HCT-116/L-OHP cells were seeded into 96-well plates (3 × 10^3^ cells per well) for 24 h at 37°C in a CO_2_ incubator. Cells were treated with a range of concentrations of curcumin (0, 5, 10, 20, 30, and 40 *μ*mol/L) to a final volume of 200 *μ*L. After 48 h incubation, the culture medium was discarded and cells were treated with 200 *μ*L of culture medium containing 10 *μ*L of CCK-8 reagent (Japan Tongren Institute of Chemistry) for 2 h. The absorbance of each well was measured on a microplate reader at 450 nm.

### 2.4. Cell Apoptosis Assays

**Figure 1:**
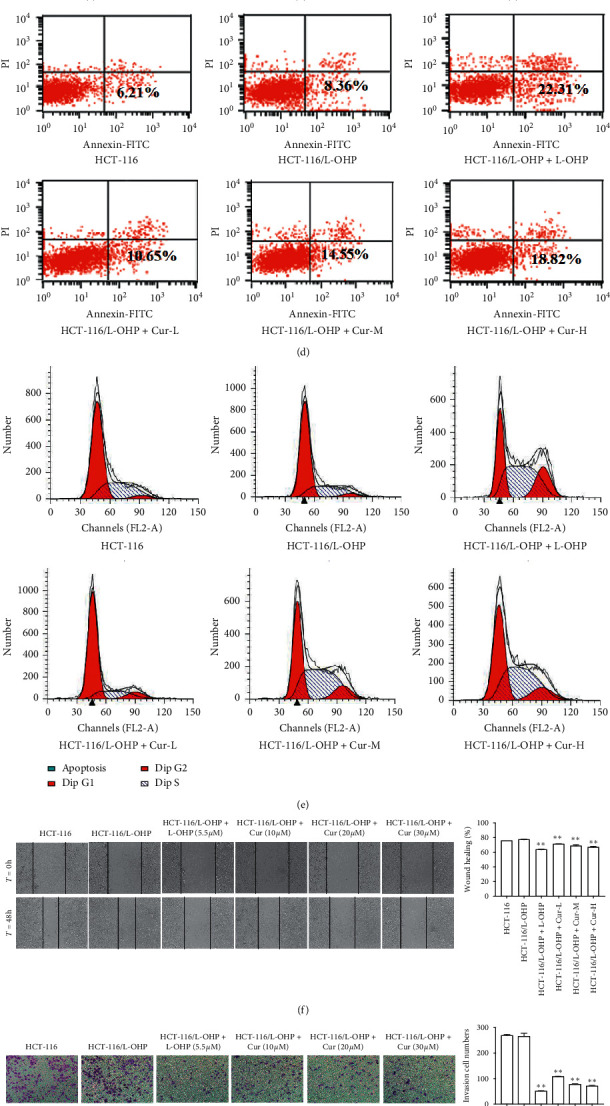
Effect of curcumin on apoptosis, cell cycle, migration, and invasion of HCT-116/L-OHP cells. (a) Structure of curcumin. (b) Effect of L-OHP on proliferation inhibition rate of HCT-116 cells and HCT-116/L-OHP cells (mean ± SEM, *n* = 3). (c) Effect of curcumin on survival rate of HCT-116 cells and HCT-116/L-OHP cells at different concentrations (mean ± SD, *n* = 3). (d) Flow cytometry for the effect of curcumin on apoptosis of HCT-116/L-OHP cells. (e) Effect of curcumin on the cell cycle of HCT-116/L-OHP by flow cytometry. (f) Migration effect of curcumin on HCT-116/L-OHP cells after 48 h incubation. (g) Invasion effect of curcumin on HCT-116/L-OHP cells after 48 h incubation. The data were presented in mean ± SD, *n* = 3 versus control group, ^*∗*^*p* < 0.05, ^*∗∗*^*p* < 0.01, ^*∗∗∗*^*p* < 0.001.

Parental and drug-resistant cells were seeded into culture dishes and treated with a range of concentrations of curcumin (Figure 1(a)) (Sigma-Aldrich, St. Louis, MO, USA): (a) HCT-116 group (control group), (b) HCT-116/L-OHP (model group), (c) HCT-116/L-OHP (L-OHP 5.5 *μ*M) group, (d) HCT-116/L-OHP (curcumin 10 *μ*M) group, (e) HCT-116/L-OHP (curcumin 20 *μ*M) group, and (f) HCT-116/L-OHP (curcumin 30 *μ*M) group. Control and model groups were cultured in drug-free culture medium, whilst experimental groups were treated with a range of concentrations of curcumin, in the culture media for a further 48 h. Cells were then collected, washed in ice-cold PBS, and resuspended in 300 *μ*L of binding buffer containing 5 *μ*L of annexin V-FITC (Shanghai Biyuntian Biotechnology Co., Ltd.) for 15 min in the dark. PI (5 *μ*L) was then added for 5 min and the rates of apoptosis were assessed by flow cytometry through the addition of 200 *μ*L of binding buffer.

### 2.5. Cell Cycle Distribution Assays

Treated cells were collected by centrifugation and washed twice in PBS. Cells were fixed in 70% ethanol at 4°C for 30 min and stained with PI for 30 min in the dark. Cell cycle distribution was detected by flow cytometry.

### 2.6. Cell Migration Assay

HCT-116, HCT-116/L-OHP, and HCT-116/L-OHP + miR-409-3p siRNA cells were seeded in 6-well plates with 1 × 10^6^ cells/well. After 24 hours of culture, scratches were made and photos were taken. Curcumin and L-OHP were given, respectively. After 48 hours of culture, photos were taken for analysis.

### 2.7. Cell Invasion Assay

Prepare a cell suspension with a concentration of 2.5 × 10^5^ cells/mL in serum-free medium, add 200 *μ*L of cell suspension to the upper chamber of the chamber (coated with Matrigel), and add the lower chamber to complete culture with 10% FBS. After 48 hours of incubation, the different drugs were prepared with 4% paraformaldehyde, stained with 0.1% crystal violet, and photographed for analysis.

### 2.8. Double Luciferase Reporter Gene Experiment and Coimmunoprecipitation Experiment

Luc-Pair™ Duo-Luciferase Assay Kit 2.0 was provided by GeneCopoeia, Inc. (USA). Immunoprecipitation Kit Dynabeads® Protein G was purchased from life technology (USA). The experimental procedure was carried out according to the kit instructions.

### 2.9. Transfection of the miR-409-3p Inhibitor into Drug-Resistant HCT-116/L-OHP

The miR-409-3P inhibitor was synthesized by Guangzhou Ruibo Biotechnology Co., Ltd. HCT-116/L-OHP cells in logarithmic growth phase were seeded into 6-well plates (4 × 10^5^ cells per well) for 24 h and transfected with miR-409-3P inhibitors using Lipofectamine 2000 transfection reagent (Invitrogen, Carlsbad, CA, USA). Cells were collected after 24 h and transfected drug-resistant cells were treated with curcumin or mock-treated.

### 2.10. Western Blot Analysis

To investigate the effects of curcumin on the expression of drug resistance-proteins of HCT-116/L-OHP cells, cells were divided into 6 groups: (a) HCT-116 group (control group), (b) HCT-116/L-OHP (model group), (c) HCT-116/L-OHP (curcumin 10 *μ*M) group, (d) HCT-116/L-OHP (curcumin 20 *μ*M) group, (e) HCT-116/L-OHP (curcumin 30 *μ*M) group, and (f) HCT-116/L-OHP (L-OHP 5.5 *μ*M) group. To investigate the effects of curcumin and miR-409-3p on the expression of drug resistance-proteins of HCT-116/L-OHP cells, cells were divided into 5 groups: (a) HCT-116 group (control group), (b) HCT-116/L-OHP (model group), (c) HCT-116/L-OHP + curcumin (curcumin 30 *μ*M) group, (d) HCT-116/L-OHP + miR-409-3p siRNA group, and (e) HCT-116/L-OHP + curcumin + miR-409-3p siRNA (curcumin 30 *μ*M) group. Cells cultured for 4 h were trypsinized (0.25% trypsin, Gibco) and lysed (Trizol). Total proteins were extracted by centrifugation at 12000 r/min for 10 min and resolved by SDS-PAGE electrophoresis at 90 V for 2 h. Proteins were transferred to nitrocellulose membranes at a constant current of 200 mA at 4°C. Membranes were blocked for 2 h and probed with the following primary antibodies: mouse anti-human ERCC1 (1 : 500 dilution, Abcam), mouse anti-human Bcl-2 (1 : 500 dilution, Abcam), goat anti-human GST-*π* (1 : 2000 dilution: Abcam), mouse anti-human MRP1 (1 : 50 dilution Abcam), rabbit anti-human P-gp (1 : 500 dilution, Abcam), rabbit anti-human survivin (1 : 5000 dilution, Abcam), and anti-*β*-actin (1 : 500 dilution, Abcam) antibodies at 4°C overnight. Membranes were washed three times in PBS-tween and labeled with the appropriate HRP-conjugated secondary antibodies (1 : 2000 dilution) for 2 h at room temperature. Membranes were then washed three times in PBS-T and protein bands were visualized via ECL chemiluminescence. The relative expression of each protein was quantified and normalized to *β*-actin using Image J software (v2.1.4.7).

### 2.11. Real-Time PCR Assays

Treated cells were lysed in 1 mL of Trizol reagent for RNA extraction (Table 1). RNA (1 *μ*g) was reversed transcribed into cDNA at 42°C for 60 min and 70°C for 5 min and stored at −20°C prior to use (iScript cDNA synthesis kit, Bio-Rad). cDNA templates (2 *μ*L) were then amplified: PCR conditions: predenaturation at 95°C for 5 min; denaturation at 95°C for 5 s; annealing at 60°C for 20 s; extension at 72°C for 20 s for 40 cycles; 95°C for 5 s, and 65°C for 1 min (iQ SYBR Green Supermix, Bio-Rad). The relative expression of miR-409-3p in each group was determined using the 2^−ΔΔCt^ method.

### 2.12. Statistical Analysis

Experiments were repeated 3 times and data were analyzed using SPSS24.0 software. Data comparisons between the groups were analyzed using a one-way analysis of variance (ANOVA). Pairwise comparisons were performed by single factor paired sample *t*-tests. *p* values <0.05 indicated a statistically significant difference.

## 3. Results

### 3.1. Establishment of HCT-116/L-OHP-Resistant Cell Models

We obtained a cell line that could survive in a medium containing 6 mg/L L-OHP with relatively stable growth rates and termed HCT-116/L-OHP cells. HCT-116/L-OHP cells were continually cultured in drug-free medium for 2 weeks in which no changes in drug resistance were observed upon the addition of L-OHP-containing medium. CCK-8 assays were performed to assess the inhibitory effects of L-OHP on drug-resistant cells. The results showed that the IC_50_ of the drug on parental cells was 5.70 mg/L compared to 71.68 mg/L in resistant cells (*p* < 0.01). The drug resistance index (RI) was 12.6, which was consistent with moderate drug resistance (Figure 1(b)).

### 3.2. Inhibition Rates of Curcumin

The survival rates of parent cell HCT-116 and drug-resistant HCT-116/L-OHP cells increased in the presence of low curcumin concentrations, whilst those of the parental cell line HCT-116 increased more significantly than HCT-116/L-OHP drug-resistant cell lines. Moderately high curcumin concentrations significantly inhibited the proliferation of parental and drug-resistant cells, the survival rates of which significantly decreased (*p* < 0.05) (Figure 1(c)).

### 3.3. Cell Cycle Distribution, Apoptotic Rates, Migration, and Invasion

The apoptotic rates of the model group were higher than those of the control group (*p* < 0.05). The apoptotic rates were 6.21% in the control group and 8.36% in the model group. The apoptotic rates of drug-resistant HCT-116/L-OHP cells treated with curcumin at low, medium, and high concentrations were 10.65%, 14.55%, and 18.82%, respectively, which were significantly higher than those of the model group (*p* < 0.01). The apoptotic rates increased with increasing drug concentrations (Figure 1(d)). Flow cytometry was used to detect the number of PI positive cells in HCT-116/L-OHP cells treated with curcumin in control, model, and curcumin groups. The results indicated that basal drug-resistant cells were arrested in the S and G_2_/M phases in the presence of curcumin (Figure 1(e)). Curcumin could significantly inhibit the migration and invasion of HCT-116/L-OHP cells (Figures 1(f) and 1(g)).

### 3.4. Curcumin Regulates the Expression of Drug Resistance-Related Proteins

**Figure 2:**
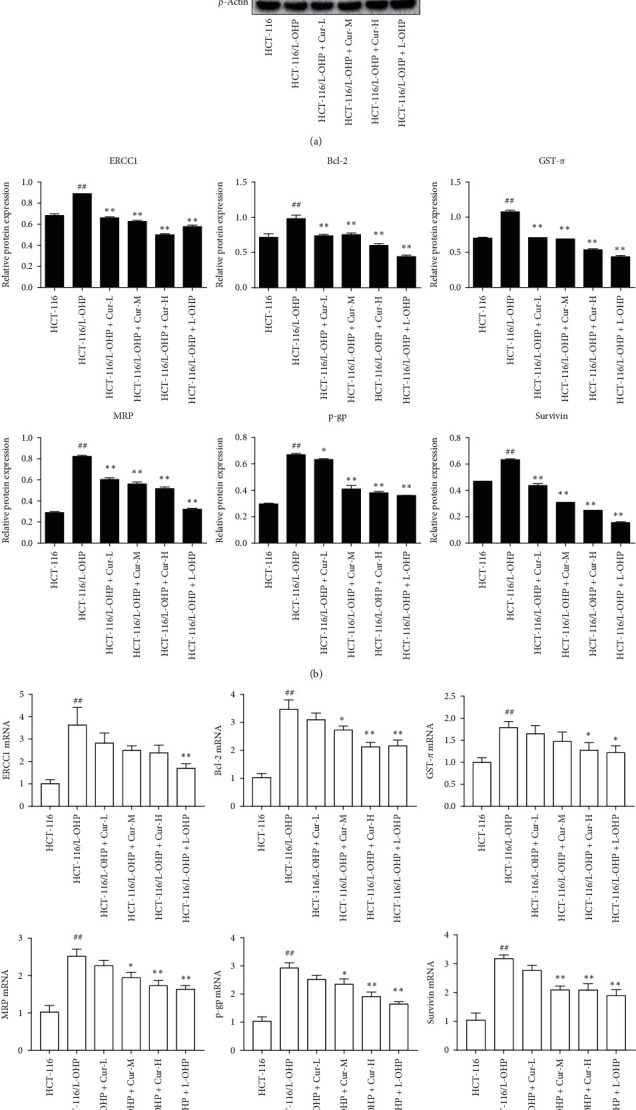
Effects of curcumin on the protein and mRNA expression of ERCC1, Bcl-2, GST-*π*, MRP, P-gp, and survivin in HCT-116/L-OHP cells. (a) ERCC1, Bcl-2, GST-*π*, MRP, P-gp, and survivin protein expression electrophoresis. (b) Effect of curcumin on protein expressions of ERCC1, Bcl-2, GST-*π*, MRP, P-gp, and survivin (mean ± SD, *n* = 3). (c) mRNA expression of ERCC1, Bcl-2, GST-*π*, MRP, P-gp, and survivin versus HCT-116 group (mean ± SD, *n* = 3), ^##^*p* < 0.01 versus HCT-116/L-OHP group, ^*∗*^*p* < 0.05, ^*∗∗*^*p* < 0.01.

Western blot analysis was used to detect the expression of ERCC1, P-gp, MRP, survivin, GSTs, and Bcl-2 in HCT-116/L-OHP-resistant cells before and after curcumin and L-OHP treatment (Figures 2(a)-2(b)). The expression of ERCC1, Bcl-2, GST-*π*, MRP, P-gp, and survivin significantly increased in the model group compared to the control group (*p* < 0.01). Following curcumin (10, 20, and 30 *μ*M) or L-OHP (5.5 *μ*M) treatment, the expression of each drug-resistant related protein was significantly lower than that of the model group (*p* < 0.01).

### 3.5. Curcumin Regulates the Expression of Drug Resistance-Related Genes

Compared to the control group, the mRNA levels of ERCC1, Bcl-2, GST-*π*, MRP, P-gp, and survivin in the model group were significantly higher (*p* < 0.01, Figure 2(c)). After curcumin treatment, the expression of each target gene decreased in a concentration-dependent manner. The expression of ERCC1 mRNA in curcumin low, medium, and high groups was lower than that of the model group (*p* < 0.05), particularly in the medium and high concentration groups (*p* < 0.01). Compared to the model group, the expression of GST-*π* in the high curcumin group significantly decreased (*p* < 0.01). Compared to the model group, the expression of Bcl-2, MRP, P-gp, and survivin at low, medium, and high curcumin concentrations significantly decreased (*p* < 0.01). Furthermore, the expression of ERCC1, Bcl-2, GST-*π*, MRP, P-gp, and survivin at L-OHP group significantly decreased compared to the model group (Figure 2(c)).

### 3.6. Identifying the Candidate miRNAs (miR-409-3p) That Regulate ERCC1 Gene Expression

**Figure 3:**
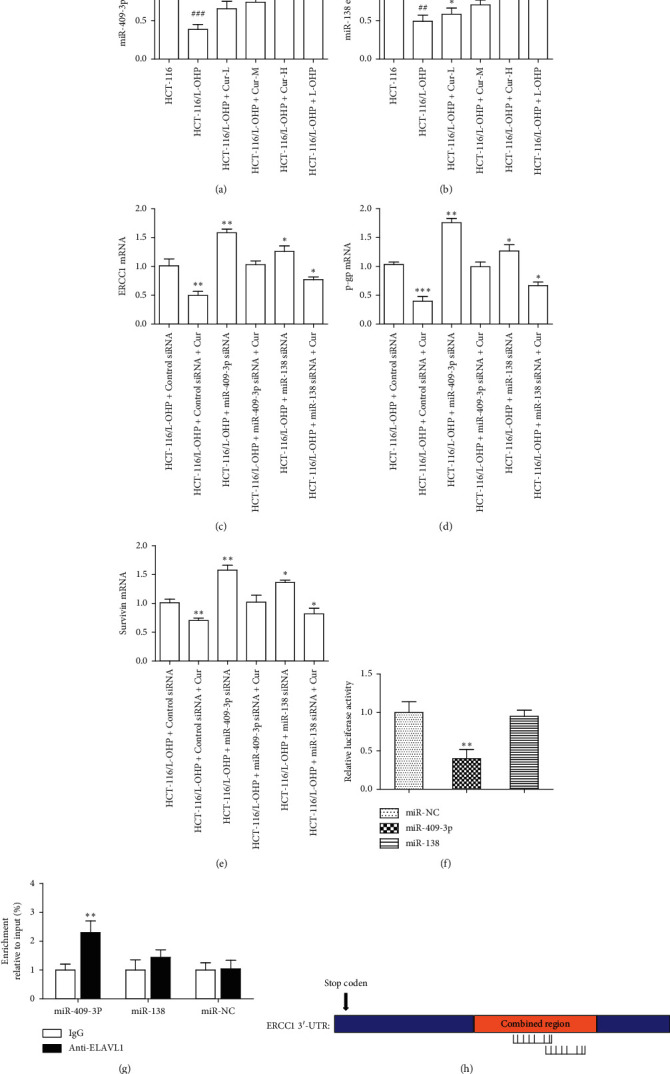
MiR-409-3p can specifically bind to ERCC1 and inhibit the mRNA expression of ERCC1. (a)-(b) The expression of miR-409-3p and miR-138 in different group. (c)–(e) ERCC1, P-gp, and survivin mRNA expression in miR-409-3p or miR-138 siRNA treatment group. (f)-(g) Double luciferase reporter gene test and immunoprecipitation test were used to compare the binding ability of miR-409-3p/miR-138 and ERCC1. (h) Bioinformatics analysis of the binding of miR-409-3p to ERCC1. The data were presented in mean ± SD, *n* = 3 versus HCT-116 group, ^##^*p* < 0.01, ^###^*p* < 0.001 versus control group, ^*∗*^*p* < 0.05, ^*∗∗*^*p* < 0.01, ^*∗∗∗*^*p* < 0.001.

Compared to HCT-116 cells, miR-409-3p and miR-138 expression in HCT-116/L-OHP cells significantly decreased (*p* < 0.01). Following curcumin treatment (10, 20, 30 *μ*M), their expression in HCT-116/L-OHP increased (*p* < 0.05) in a concentration-dependent manner and their expression in L-OHP (5.5 *μ*M) group also increased (*p* < 0.01) compared to HCT-116/L-OHP cells (Figures 3(a)-3(b)). The mRNA expression levels of ERCC1, p-gp, and survivin were upregulated in HCT-116/L-OHP cells treated with mir-409-3p siRNA and miR-138 siRNA, respectively. After curcumin was given, the expression levels of ERCC1, p-gp, and survivin would be reduced again (Figures 3(c)–3(e)). The results of double luciferase reporter gene test and immunoprecipitation test indicated that miR-409-3p and ERCC1 have high binding ability (Figures 3(f)-3(g)). Bioinformatics analysis revealed that the 3′-UTR of the ERCC1 gene contains a miR-409-3p binding site (Figure 3(h)) suggesting that miR-409-3P targets the ERCC1 gene. miR-409-3p was therefore selected as a candidate miRNA.

### 3.7. Effects of miR-409-3p and Curcumin on Cell Cycle Distribution, Apoptotic Rates, Migration, and Invasion

**Figure 4:**
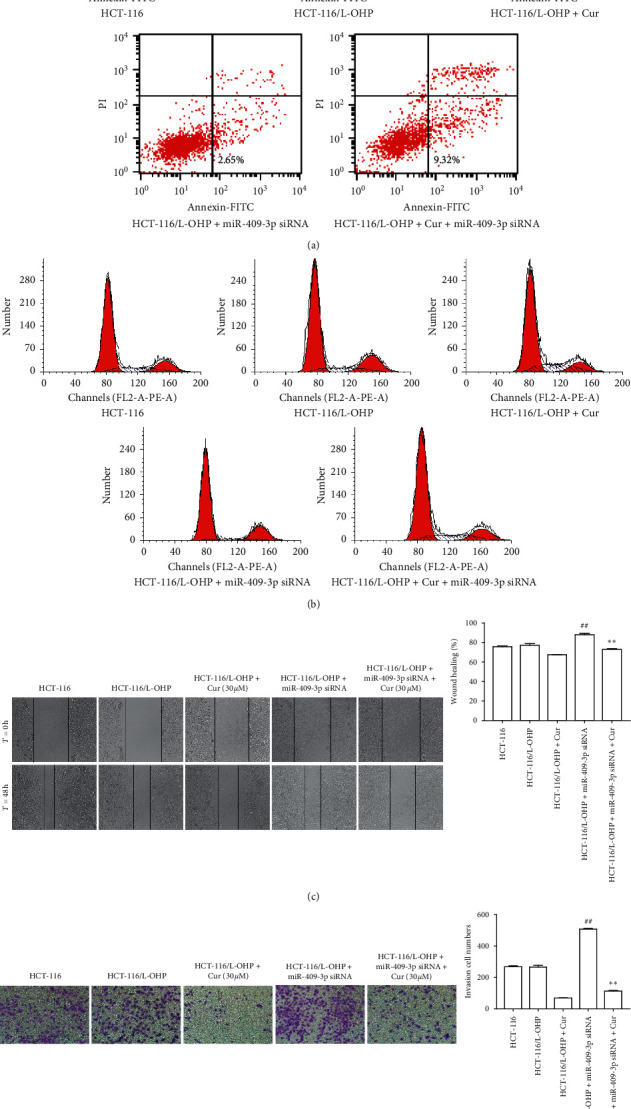
Effect of curcumin and miR-409-3p on apoptosis, cell cycle, migration, and invasion of HCT-116/L-OHP cells. (a) Flow cytometry for the effect of curcumin and miR-409-3p on apoptosis of HCT-116/L-OHP cells. (b) Effect of curcumin and miR-409-3p on the cell cycle of HCT-116/L-OHP by flow cytometry. (c) Migration effect of curcumin and miR-409-3p on HCT-116/L-OHP cells after 48 h incubation. (d) Invasion effect of curcumin and miR-409-3p on HCT-116/L-OHP cells after 48 h incubation. The data were presented in mean ± SD, *n* = 3 versus HCT-116/L-OHP group, ^##^*p* < 0.01 versus HCT-116/L-OHP + Cur group, ^*∗∗*^*p* < 0.01.

After curcumin treatment, the apoptotic rate of HCT-116/L-OHP cells increased. However, after miR-409-3p siRNA treatment, the apoptotic rate of HCT-116/L-OHP cells decreased. And then, given curcumin, the apoptotic rate of HCT-116/L-OHP cells would increase again (Figure 4(a)). The results of flow cytometry test indicated that HCT-116/L-OHP with miR-409-3p siRNA treatment were arrested in the S and G2/M phases in the presence of curcumin (Figure 4(b)). Compared with HCT-116/L-OHP group, after the treatment of miR-409-3p siRNA, the migration and invasion ability of HCT-116/L-OHP cells increased. However, curcumin could significantly inhibit their migration and invasion (Figures 4(c) and 4(d)).

### 3.8. Effects of miR-409-3p and Curcumin on the Protein Expression of ERCC1, Bcl-2, GST-*π*, MRP, P-gp, and Survivin in HCT-116/L-OHP Cells

**Figure 5:**
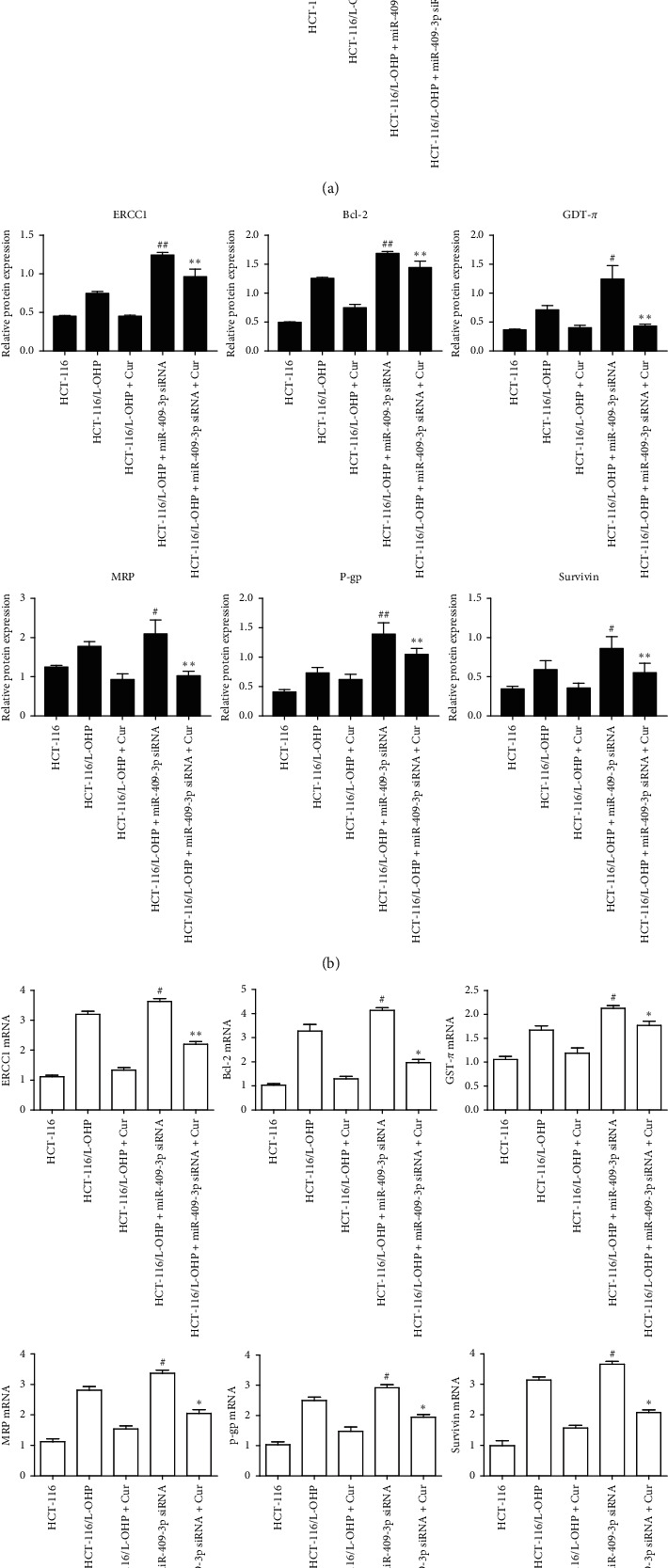
Effects of curcumin and miR-409-3p on the protein and mRNA expression of ERCC1, Bcl-2, GST-*π*, MRP, P-gp, and survivin in HCT-116/L-OHP cells (mean ± SD, *n* = 3). (a) ERCC1, Bcl-2, GST-*π*, MRP, P-gp, and survivin protein expression electrophoresis. (b, c) Effect of curcumin and miR-409-3p on protein and mRNA expressions of ERCC1, Bcl-2, GST-*π*, MRP, P-gp, and survivin versus HCT-116/L-OHP group, ^#^*p* < 0.05, ^##^*p* < 0.01 versus HCT-116/L-OHP + Cur group, ^*∗*^*p* < 0.05, ^*∗∗*^*p* < 0.01.

Compared to parental cell HCT-116 cells, the expression of ERCC1, Bcl-2, GST-*π*, MRP, P-gp, and survivin in drug-resistant HCT-116/L-OHP cells significantly increased. Compared to HCT-116/L-OHP cells, the expression of ERCC1, Bcl-2, GST-*π*, MRP, P-gp, and survivin in HCT-116/L-OHP cells transfected with miR-409-3p inhibitors further increased (*p* < 0.01). The expression of ERCC1, Bcl-2, GST-*π*, MRP, P-gp, and survivin in HCT-116/L-OHP cells decreased after curcumin treatment in the presence or absence of the miR-409-3p inhibitor (30 *μ*M) (*p* < 0.01) (Figures 5(a)-5(b)).

### 3.9. Effects of miR-409-3p and Curcumin on the mRNA Expression of ERCC1, Bcl-2, GST-*π*, MRP, P-gp, and Survivin in HCT-116/L-OHP Cells

Compared to parental HCT-116 cells, the mRNA expression of ERCC1, Bcl-2, GST-*π*, MRP, P-gp, and survivin in the drug-resistant HCT-116/L-OHP cells significantly increased. Compared to drug-resistant HCT-116/L-OHP cells, the *mRNA* expression of ERCC1, Bcl-2, GST-*π*, MRP, P-gp, and survivin in drug-resistant HCT-116/L-OHP cells transfected with inhibitors of miR-409-3p further increased (*p* < 0.01). The mRNA expression of ERCC1, Bcl-2, GST-*π*, MRP, P-gp, and survivin in HCT-116/L-OHP cells in the presence and absence of miR-409-3p inhibitors significantly decreased after curcumin treatment (30 *μ*M) (*p* < 0.01) (Figure 5(c)).

## 4. Discussion

In recent years, the incidence and mortality of colorectal cancer has increased on a yearly basis. Patients in advanced disease stages are treated by chemotherapy, but drug resistance reduces its efficacy. The drug resistance mechanisms of tumor cells are mainly related to increased drug pump efflux, drug detoxification, changes in apoptosis-related pathway, and alterations in DNA repair ability. The aim of this study was to identify the mechanisms of the reversal of drug resistance in HCT-116/L-OHP cells treated with curcumin. We demonstrated that the inhibition rates of drug-resistant cell lines at equivalent drug concentrations were significantly lower than those of sensitive strains. This indicated that the drug-resistant cell lines were successfully established. Compared to L-OHP, curcumin shows promising inhibitory effects on drug-resistant cells in a concentration-dependent manner. Therefore, curcumin can reverse the resistance of HCT-116 cells to L-OHP.

The expression of ERCC1 in the HCT-116/L-OHP resistant cells was significantly higher than that of parental HCT-116 sensitive cells. Similar data were observed upon assessment of the expression of Bcl-2, GST-*π*, MRP, P-gp, and survivin. Bcl-2 is a key regulator of cell apoptosis. High Bcl-2 levels can inhibit cell apoptosis, accelerate cell growth promote tumor malignancy [17]. GST-*π* is a second-phase metabolic enzyme that is highly expressed in many human cancers. It binds to GSH (glutathione) and is secreted by multidrug resistance-related proteins to reduce the cytotoxicity of chemotherapy drugs [18]. MRPs recognize chemotherapeutic drugs to which they couple to reduce intracellular drug concentrations thus promoting resistance [19]. P-gp is an energy-dependent transporter that binds to drugs and ATP to supply the energy for chemotherapeutic drug efflux [20]. Survivin is an antiapoptotic protein expressed in a variety of tumors that promotes the proliferation of tumor cells, resulting in tumor resistance [21]. The increased expression of Bcl-2, GST-*π*, MRP, P-gp, and survivin suggests an increase in HCT-116/L-OHP resistance.

The main apoptotic mechanism(s) of L-OHP is/are covalent binding with the base groups in DNA chains to form Pt-DNA complexes in the nucleus, which lead to DNA interchain or intrachain cross-links, resulting in DNA duplication and transcriptional damage. Nucleotide excision repair (NER) is the main pathway by which tumor cells improve DNA repair ability. Excision repair cross-complementing gene 1 (ERCC1) is key to the nucleotide excision repair process. When ERCC1 is overexpressed, the DNA repair ability of tumor cells increases and their sensitivity to platinum drugs decreases [22]. Previous studies have shown that single nucleotide polymorphisms of ERCC1 contribute to the susceptibility of colorectal cancer patients to L-OHP [16]. The results of this study confirmed that the expression of ERCC1 in HCT-116/L-OHP cells was abnormally increased, as were the expression of Bcl-2, GST-*π*, MRP, P-gp, and survivin, indicating that the resistance of colorectal cancer cells to L-OHP is mediated through ERCC1.

MicroRNAs (miRNAs) are noncoding small RNAs 18–25 nucleotides in length. miRNAs play an important role in the regulation of cell proliferation, differentiation, and apoptosis [23]. miRNAs specifically bind to target genes in the 3′UTR to regulate gene expression and protein synthesis [24]. It has been shown that the 3′-UTR of ERCC1 is closely related to the chemosensitivity of L-OHP. miRNAs regulate the transcription and translation of ERCC1 and participate in tumorigenesis. In this study, we found that miR-409-3P is a candidate miRNA that regulates ERCC1 through using bioinformatics analysis. RT-PCR analysis showed that the expression of miR-409-3p in resistant HCT-116/L-OHP cells was significantly lower than that of parent HCT-116 cells, suggesting that the downregulation of miR-409-3p expression is related to the drug resistance of colorectal cancer cells. Upon comparison of HCT-116, HCT-116/L-OHP, and HCT-116/L-OHP + miR-409-3p siRNA groups, the apoptotic rates decreased and the expression of drug resistance-related genes increased, indicating that the degree of resistance was in the order HCT-116 < HCT-116/L-OHP < HCT-116/L-OHP + miR-409-3p siRNA. The resistance of the cells also negatively correlated with the expression of miR-409-3p suggesting it mediates the drug resistance of HCT-116/L-OH cells. The downregulation of miR-409-3p expression led to the upregulation of ERCC1 expression, which enhanced the expression of the drug resistance associated proteins Bcl-2, GST-*π*, MRP, P-gp, and survivin.

Curcumin is a plant polyphenol (C_21_H_20_O_6_) [25] with a range of pharmacological effects including anti-inflammatory and antitumor activity and the reversal of cancer cell drug resistance [10]. The results suggested that the expression of ERCC1 decreases with increased curcumin concentrations and that the expression of Bcl, GST-*π*, MRP, P-gp, and survivin decreased after curcumin treatment. This indicated that curcumin can reverse drug resistance by inhibiting the expression of ERCC1 and regulating the expression of drug resistance-related proteins. The low expression of miR-409-3p in human colon cancer resistant cells may therefore promote drug resistance. Its mechanism of action is to upregulate the expression of ERCC1 and promote the expression of Bcl-2, GST-*π*, MRP, P-gp, and survivin. Curcumin reverses L-OHP resistance in human colon cancer cells. The mechanism of this effect may be mediated through ERCC1 through the upregulation of microRNA-409-3p in drug-resistant cells, thus decreasing the expression of the aforementioned drug-resistant proteins. This provides an experimental rationale to enhance colon cancer cell killing during L-OHP clinical therapy.

## 5. Conclusions

In conclusion, curcumin can reverse the drug resistance of the human colon cancer resistant cell line HCT-116/L-OHP. Its mechanism of action related to curcumin regulates ERCC1 expression and enhances L-OHP sensitivity through its effects on miR-409-3p. This reduces the secretion of L-OHP from colorectal cancer cells increasing their sensitivity to the drug. Curcumin has the advantage of low toxicity, low cost, and high availability. It therefore represents a possible drug resistance reversal agent for colon cancer in future clinical practice.

## Figures and Tables

**Table 1:** Primer sequences.

Primer		Primer sequence (5′-3′)	Length/bp
ERCC1	Forward primer (5′ ⟶ 3′)	ATGTCTGACCACCGTGAA	188
	Reverse primer (5′ ⟶ 3′)	TGTTCCAGAGATCCAAATGTG	
Bcl-2	Forward primer (5′ ⟶ 3′)	GCCTTCTTTGAGTTCGGTG	83
	Reverse primer (5′ ⟶ 3′)	AGTCATCCACAGGGCGAT	
GST-*π*	Forward primer (5′ ⟶ 3′)	CAGGAGGGTCACTCAAAG	437
	Reverse primer (5′ ⟶ 3′)	CAGGTTGTAGTCAGCGAAG	
MRP1	Forward primer (5′ ⟶ 3′)	GGCATCTCAGCAACTCGTCTT	250
	Reverse primer (5′ ⟶ 3′)	ATTAGCTTCCACGTCTCCTCCTT	
p-gp (MDR1)	Forward primer (5′ ⟶ 3′)	AGCTCATCGTTTGTCTACAGTTCG	403
	Reverse primer (5′ ⟶ 3′)	TCCACGGACACTCCTACGAGT	
Survivin	Forward primer (5′ ⟶ 3′)	AAGAACTGGCCCTTCTTGGA	313
	Reverse primer (5′ ⟶ 3′)	CAACCGGACGAATGCTTTT	
*β*-Actin	Forward primer (5′ ⟶ 3′)	CATGTACGTTGCTATCCAGGC	268
	Reverse primer (5′ ⟶ 3′)	CTCCTTAATGTCACGCACGAT	

## Data Availability

The data used to support the findings of this study are available from the corresponding author upon request.
